# Do Student Samples Provide an Accurate Estimate of the General Public?

**DOI:** 10.1371/journal.pone.0168354

**Published:** 2016-12-21

**Authors:** Paul H. P. Hanel, Katia C. Vione

**Affiliations:** School of Psychology, Cardiff University, Cardiff, United Kingdom; University of Vienna, AUSTRIA

## Abstract

Most psychological studies rely on student samples. Students are usually considered as more homogenous than representative samples both within and across countries. However, little is known about the nature of the differences between student and representative samples. This is an important gap, also because knowledge about the degree of difference between student and representative samples may allow to infer from the former to the latter group. Across 59 countries and 12 personality (Big-5) and attitudinal variables we found that differences between students and general public were partly substantial, incoherent, and contradicted previous findings. Two often used cultural variables, embeddedness and intellectual autonomy, failed to explain the differences between both groups across countries. We further found that students vary as much as the general population both between and within countries. In summary, our results indicate that generalizing from students to the general public can be problematic when personal and attitudinal variables are used, as students vary mostly randomly from the general public. Findings are also discussed in terms of the replication crisis within psychology.

## Introduction

Student samples are extremely common in psychological and cross-cultural studies due to the facility of recruitment, lower cost of administration, and assumed lower response bias [1]. In cross-cultural research for example, students are thought to reduce the variability in the sample due to differences in education levels [2], and were found to give a moderately good estimate of representative or teachers samples [3,4]. However, many concerns are raised in using student samples for psychological studies regarding issues of representativeness, generalizability, and comparability of results [5–7]. For example, Greenfield [8] argued that differences between socioeconomic groups are larger than differences between countries. As students have usually high socioeconomic status, this further challenges the generalizability of data from students. Indeed, students are, on average, more homogeneous than non-student participants, as a large second-order meta-analysis revealed [9]. Importantly, in this study the non-student samples were often only another subgroup from the general public (e.g., housewifes).

To the best of our knowledge, no study has systematically compared students with the general public across many countries. For example, are students indeed more homogeneous across countries and is it legitimate to generalize from student samples to the general public? In the present paper we intend to explore potential differences between student and representative samples and to shed more light on potential predictors of the difference between student and representative samples: The mere existence of such differences is not problematic as long as they can be systematically predicted. We focus on variables which are important in personal and social psychology: Five personality variables (the Big-5 dimensions) and seven attitudinal variables such as moral attitudes or respect towards the elderly.

As predictors of the differences between students and the general public we use two important variables in cross-cultural research: collectivism or embeddedness, and individualism or autonomy [10]. For example, people in autonomous countries prefer to stand out and to follow their own goals [10]. This would suggest that students in autonomous countries are more likely to follow their own goals than students in embedded countries, who, in contrast, will adopt the goals of the general public. Therefore, we expect that the difference between students and the general public vary as a function of how autonomous or embedded a society is.

The present study further investigates some under explored issues. Specifically, we aim to compare students with the general public from 59 countries across 12 variables, expecting that students are, in general, more homogeneous than the general public, both across countries (Hypothesis 1a) and also within countries (Hypothesis 1b). Furthermore, we aim to explain the difference between students and the general public as well as the variability within student samples. We expect that the difference between students and the general public is larger in more autonomous countries, as there is less pressure to adjust one’s own personality, attitudes and views along with the general population (Hypothesis 2a). With the reverse true for embeddedness (Hypothesis 2b), because of the large negative correlation with autonomy [10]. In line with this argument, we also expect that within-student variability is greater in autonomous countries as there is more acceptance of individual development (Hypothesis 3a). With the reverse true for embeddedness (Hypothesis 3b). This view is also supported by the finding that, across 80 countries, autonomy was strongly and positively correlated with income and democratization [10]. In summary, we are interested in patterns of variability, not mean differences.

Hypotheses 2a and 2b require some further evaluations. Based on previous research we further assumed that for several variables the sign of the difference is the same across countries. For example, a large cross-sectional study found systematic personality (Big-5) differences between younger and older participants [11]. Younger participants scored higher on neuroticism and openness to new experiences, but lower on agreeableness and conscientiousness compared to older participants. No relation was found between age and extraversion. Hence, we assumed that all student samples score higher on neuroticism and openness to new experiences but lower on agreeableness and conscientiousness. However, we expect that the magnitude of the difference between students and the general public is larger in countries higher in intellectual autonomy.

The present study is also important from another point of view. Recently, it was found that more than half of the studies within psychology could not be replicated [12]. However, this reproducibility project was criticized for not taking contextual differences into account, as some studies were replicated in another country, which may have partly caused the low reproducibility rate [13]. This is in line with calls for studies to explore whether effects vary across contexts and if so, how [14]. If we find systematic differences between students and the general public (Hypotheses 2a and 2b) this would allow us to make specific predictions about when a study, which compares students and the general public, can be replicated in a different context. This may also help to explain post-hoc the variance in effect sizes of large scale reproducibility projects [12,15]: The variance of effect size in more embedded countries or regions should be smaller.

## Method

### Participants

We used the most recent version of the World Values Survey (WVS, 6^th^ round) at the time of data analysis, which includes samples of 86,272 general public participants (51.20% female) from 60 countries, with a mean age of 41.68 years (*SD* = 16.58). Data were retrieved from http://www.worldvaluessurvey.org (data set released on 16 April 2015). Data from Argentina were excluded as the employment status was missing. Of the remaining 84,737 participants, 6,352 reported being students (*M* = 105.90 per country, *SD* = 79.24), leaving 78,385 non-students (*M* = 1328.22, *SD* = 442.45). Non-surprisingly, across all 59 countries the general public was almost twice as old (*M* = 43.23, *SD* = 16.10) as the students (*M* = 21.98, *SD* = 6.64, *d* = 2.74). Also, students were on average more educated than the general public, *t*(8301.14) = 51.42, *d* = 0.68. Because the non-students vary strongly with regard to factors such as their income, education, employment status, and political attitudes within each country, we refer to the non-student samples as general public.

### Materials

In total, we used two independent and twelve dependent variables. The independent variables were embeddedness as an estimator of collectivism, and intellectual autonomy as a proxy for individualism [10]. Both are part of Schwartz’ [10] cultural value orientation model (CVO). To measure the cultural value orientations, participants were asked to indicate on a 9-point scale how much each of the 56 values is a guiding principle in their life. Examples include “FREEDOM (freedom of action and thought)” for intellectual autonomy and “OBEDIENT (dutiful, meeting obligations)” for embeddedness. The 56 values were combined to form seven CVOs, including embeddedness and intellectual autonomy [10]. The data were centered on an individual bases, i.e., the individual overall mean for the 56 items was subtracted from each participant response. This was done to correct for individual scale use tendencies and to investigate the relative importance of CVOs [10,16]. The CVOs were made publicly available by Schwartz [17].

The dependent variables were chosen out of the wide range of variables available in the WVS, based on good reliabilities (αs ≥ .70). We used the items of the Morally Debatable Scale [18], which were divided into three sub-scales after a principal component analysis (cf. [19]): attitudes towards liberal personal-sexual behaviors (7 items, α = .89), dishonest-illegal behaviors (5 items, α = .83), and domestic violence (3 items, α = .78). For example, participants were asked how justifiable they find homosexuality or abortion (personal-sexual behaviors), stealing property or accepting a bribe (dishonest-illegal), and a man beating his wife (domestic violence). Responses were given on a 10 point scale, ranging from 1 (never justifiable) to 10 (always justifiable).

The other variables were trust in strangers (e.g., “How much do you trust people of another religion?”, 3 items, α = .79), understanding of democracy (e.g., is “Governments tax the rich and subsidize the poor” an essential characteristic of democracy?, 6 items, α = .74), confidence in political institutions (e.g., “How much trust do you have in the parliament?”, 6 items, α = .87), and perceived respect of own society towards elderly (e.g., “People over 70 are viewed with respect by my society”, 3 items, α = .71).

Additionally, we included the ten-item personality inventory which measures each of the so called Big-5 dimensions of personality with two items [20]. The five dimensions are extraversion (e.g., “I see myself as someone who is outgoing, sociable”), agreeableness (e.g., “I see myself as somene who is generally trusting”), conscientiousness (e.g., “I see myself as someone who does a thorough job”), neuroticism (e.g., “I see myself as someone who gets nervous easily”), and openness to new experiences (e.g., “I see myself as someone who has few artistic interests” [reversed coded]). One item of each dimension was reversed coded. Responses were given on a 5 point Likert scale ranging from 1 (disagree strongly) to 5 (agree strongly). As the measure was designed to measure five broad dimensions with only two items, reliabilities were as expected [21] low (αs ≤ .39). Nevertheless, other psychometric qualities such as convergent validity or test-retest reliabilities were found to be good [20]. Data for this measure was available for 25 countries.

## Results

We have made the transformed data available (i.e., the summary statistics for all 59 countries; see S1 Data) for Hypotheses 2a and 2b along with the R script (S1 File) used to compute them. The other steps of the analyses (i.e., the tests for Hypotheses 1 and 3) can be reproduced with the R script and the original WVS data, which can be obtained from www.worldvaluessurvey.org.

To test Hypothesis 1a –are students more homogeneous compared to the representative sample across countries?–, we first computed separate medians for each group, country, and variable, as some variables were skewed. Variation of the medians within the students across the 59 countries was then compared with the variation within the general public, using a Levene test for each of the 7 DVs. This tested the null hypothesis that the variances of the medians do not differ between both groups. For example, the standard deviation of the medians for the student samples for trust in science across all 59 countries was 0.31, and for the general public samples 0.32 (see Table 1, columns 2–3 for *SD*s). Contrary to Hypothesis 1a, none of the Levene tests reached statistical significance, neither for the Big-5 (all *F*s[1, 48] < 2.70, *p*s > .10) nor the seven attitudinal variables (all *F*s[1, 116] < 1.05, *p*s > .30), indicating that the variation of the medians was approximately the same for students and the general public across all 59 countries. To test Hypothesis 1b –are students more homogeneous compared to the representative sample within countries?–, we computed the within-country variability (standard deviation) separate for each of the two groups and each country and compared the *SD*s using independent sample t-tests. None of the twelve t-tests reached statistical significance, neither for the Big-5 dimensions (*t*s[23] ≤ |0.80|, *p*s > .43, and *d*s ≤ |.22|) nor seven attitudinal variables (*t*s[57] ≤ |1.97|, *p*s > .05, and *d*s ≤ |.36|). Because the medians may not be normal distributed, we additionally computed twelve Wilcoxon rank sum test. However, results remained the same (all *p*s > .06). This indicates that students were on average as heterogeneous as the general public both between and within countries.

**Table 1:** Zero-order correlation coefficient of Cohen’s d (student vs. general public) and within country variance of students with predictors.

				Cohen’s d		Variance	
	SD Stud	SD GP	$\bar{d}$	Embeddedness	Intellectual Autonomy	Embeddedness	Intellectual Autonomy
Extraversion	0.25	0.20	0.10	-.22	.37	-.65**	.61*
Agreeableness	0.40	0.54	-0.08	-.01	.14	.63**	-.52*
Conscientiousness	0.67	0.70	-0.25	-.51*	.38	.06	-.07
Neuroticism	0.29	0.25	0.03	-.27	.22	-.31	.43
Openness	0.36	0.32	-0.02	-.13	.11	-.52*	.56*
MA: Domestic violence	0.89	0.92	0.06	-.11	.14	.18	-.21
MA: Dishonest-illegal behavior	0.75	0.73	0.18	-.47**	.49**	.16	-.20
MA: Personal-sexual behavior	1.74	1.56	0.21	-.10	.07	-.35*	.24
Trust in strangers	0.31	0.32	-0.04	-.11	.18	.07	-.17
Understanding of democracy	0.81	0.77	-0.03	.30	-.22	.38*	-.40**
Confidence in pol. Institutions	0.40	0.41	-0.03	-.20	.26	.20	-.22
Respects towards elderly	0.38	0.38	-0.02	-.02	-.08	.23	-.31

SD: Standard deviation, Stud: Students, GP: General public, $\bar{d}$: mean standardized difference of students to general public (if d > 0: Students score higher); *N* of countries for SD (columns 2–3) = 25 (Big-5) and 58–59, *N* of countries (columns 5–8) = 16 (Big-5) and 39–40. Cohen’s ds were computed with pooled standard deviations.

* p < .05

** p < .01

Hypotheses 2a and 2b stated that the difference between students and the general public is greater in autonomous countries, and smaller in embedded ones, respectively. In a first step, we tested the underlying assumption of these Hypotheses. That is, the differences between students and the general public varies across countries. To test the assumption, we compared a random intercept and random slope model with a random intercept and fixed slope model. A likelihood ratio test revealed a significant difference for all twelve variables, χ^2^(2)s = 15–205, *p*s < .001, indicating that the slopes were not the same across countries. In a next step, we then compared whether intellectual autonomy and embeddedness explained some of the variation. Because it is not possible to address this question within a multi-level framework, we used an alternative approach, which is described below.

Cohen’s ds were computed as a measure of the difference between the two groups for all 59 countries, with pooled standard deviations. Cohen’s ds differed up to |.80|, but mainly between -.5 and .5, whereas the distribution of *d*s were mostly normal with a mean of around 0 (see Table 1, column 4 for the means of d). Next, we correlated the absolute value of d for each variable with the country average score for intellectual autonomy and embeddedness.

The predicted pattern of results for Hypotheses 2a and 2b was found only for moral attitudes towards dishonest-illegal (Table 1, columns 5–6). In countries with higher embeddedness values, students endorsed moral attitudes towards dishonest-illegal behaviors more similarly to the the general pulic. The reverse pattern was observed in countries high in intellectual autonomy (this is not surprising, given that embeddedness and intellectual autonomy were strongly negatively correlated, *r* = -.87). Of the remaining 22 correlations only one reached statistical significance (Table 1, columns 5–6).

We have also used the Human Developmental Index [22] and the Democracy Index [23] as independent variables. However, because of high correlations with intellectual autonomy (*r* = .70 and *r* = .64, respectively), results were very similar and are, therefore, omitted. We have further added the tightness scores [24] as an independent variables for 20 countries. This also did not change the pattern of results.

**Fig 1:**
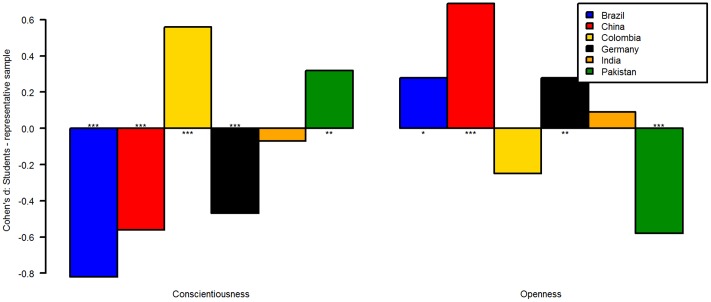
Cohen's d of student—general public comparisons for two of the Big-5 dimensions. See text for explanation. *p < .05, **p < .01, ***p < .001.

**Fig 2:**
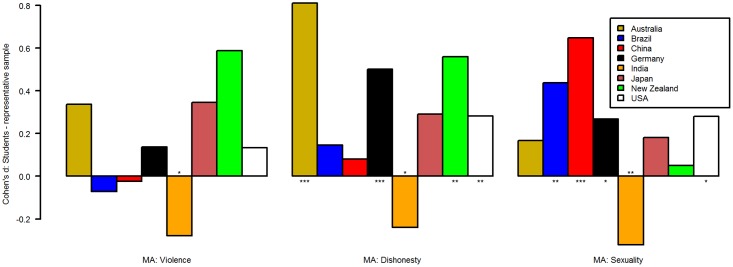
Cohen's d of student—general public comparisons for moral attitudes (MA). See text for explanation. *p < .05, **p < .01, ***p < .001.

**Fig 3:**
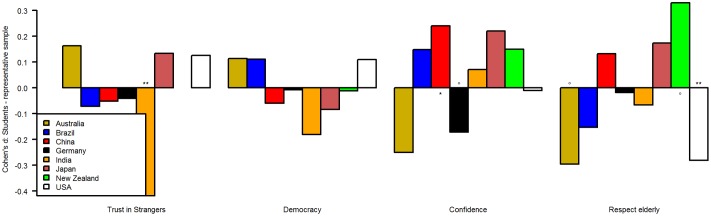
Cohen's d of student—general public comparisons for several attitudinal variables. ° < .08, *p < .05, **p < .01, ***p < .001.

Because of this failure to find a predictor for the difference between students and general public, we investigated the d-scores more closely. Within each variable, some of the ds were positive, others negative, both with and without reaching statistical significance. To give an illustration, we have selected six countries for two of the personality variables and eight countries for the remaining seven variables and plotted the d-scores for each variable (Figs 1–3). Countries were selected based on the number of participants which originate from them on average in psychological research [1] and effect size. Please note that in some countries effect sizes were larger than the ones depicted here (see data). Fig 1 depicts the d-scores for conscientiousness and openness for six countries. For example, students in Brazil, China, and Germany were less conscientious than the general public, but more in Colombia and Pakistan. In Fig 2, the d-scores for moral attitudes towards (domestic) violence, dishonest-illegal behavior, and towards personal-sexual behaviors are depicted. In Fig 3 the d-scores for trust in strangers, understanding of democracy, and perceived respect towards elderly are displayed. Some differences between the students and the general public were larger compared to others (e.g., moral attitudes towards dishonest-illegal behaviors vs. understanding of democracy). Please note that the p-values are reported for convenience only. Because this analysis was exploratory, p-values cannot be meaningfully interpreted. We did not adjust for multiple-comparisons because both the number of tests for which we would need to control and the adjustment method itself can only be arbitrarily chosen and therefore will reduce transparency. Instead, we emphasize that the within-country findings (e.g., differences between both sample types for country X) should be tested in further confirmatory studies [25].

Additionally, we explored the percentage of occasions for which the null-hypothesis was supported or the alternative hypothesis. For this, we computed the Bayes factor (BF) for all 537 student-general public comparison, as the classical frequentist approach does not allow to address the question whether the data supports the null hypothesis [26]. BFs were computed with the R package BayesFactor [27], with the default prior of r = .707. Out of the 537 computed BFs, 23 showed strong support for the null hypothesis (BF < 1/10), 299 moderate support (BF < 1/3), 97 moderate support for the alternative hypothesis (BF > 3) and 78 strong support for the alternative hypothesis (BF > 10). For the remaining 40 BFs neither support for the alternative nor null hypotheses was found (1/3 < BF < 3).

To test Hypotheses 3a and 3b –students in countries with high (vs. low) intellectual autonomy (vs. embeddedness) values are more heterogeneous—the standard deviation of students within each country was computed and correlated with the average intellectual autonomy and embeddedness score of each country. This was done for all twelve variables. This hypothesis was only partially supported for extraversion, openness, and moral attitudes towards personal-sexual behaviors. Specifically, students in countries high in embeddedness were on average more homogenous, i.e., the variance was lower. For the variables agreeableness and understanding of democracy the pattern of correlations was the opposite of the expected. The pattern of results for the remaining variables was mixed (Table 1, columns 7–8).

## Discussion

Contrary to previous findings [9] and reflections [8], students were across 59 countries and twelve variables as heterogeneous as the general public. Also, for some variables, students scored higher in some countries than the general public, although previous research has implied that students should score lower—or vice versa—because of age effects [11]. However, consistent with previous findings [9], we were neither able to explain the difference between students and the general public nor the variability of students across countries. Differences between students and the general public in some countries were positive, negative, or non-significant (cf. [6]). This challenges the claim that students are a moderately accurate estimate for the representative sample [3,28].

### Implications and limitations

Our findings have important implications for psychological research. First, we demonstrated that students vary both across and within countries as much as the general public. This means that universities do not influence personality attitudes of students or at least not in the same way across countries and/or subjects. A possible explanation for this is that the subject of study has an impact on psychological variables. For example, it was found that psychology students increasingly value benevolence over the course of three years, whereas business students increasingly value achievement and power [29]. In other words, the apparent random differences between students and general public across countries may be partly attributed to differences in the subject of the students; we did not control for the subject, as we did not have any data available for this and the average student sample size of 105 per country would have been too small to conduct subgroup analyses. Hence, this would be a possible way to go for a further cross-cultural study, to compare students from different subjects across countries.

Moreover, because students are both younger and more educated than the general public, we do not know if age or level of education—or an interaction—is responsible for the large variability within the student sample. Next, we have found that neither embeddedness nor autonomy predicted the difference between students and the general public, indicating that students in embedded countries differ as much from the general public as in autonomous countries. In other words, autonomy and embeddedness, as measured within a society, do not predict attitudes of students relative to the general public. Also, we found that students, despite being on average only half of the age, scored in some countries higher on conscientiousness and agreeableness and lower on neuroticism and openness. This partly relativizes findings from cross-sectional studies which imply that younger people score lower on conscientiousness and agreeableness but higher on neuroticism and openness [11]. Together, this further supports the claim that generalizing from students to the general public within personal and social psychology is problematic (e.g., [5,7]), at least whilst we do not know what predicts those differences. Our Bayesian analyses further supported this claim. In order to be able to generalize from the students to the general public, we would require that they do not differ. However, our Bayesian analysis revealed for only 23 out of 537 comparisons (4%) strong evidence for the null hypothesis.

To illustrate why generalizing from students to the general public is problematic, assume a researcher wants to test her hypothesis that Chinese are more open towards new experiences than Pakistani. She collects one student sample in each country and finds allegedly strong support for her hypothesis, *t*(204) = 9.84, *p* < .0001, d = 1.38 (data for this example is taken from WVS, cf. also Fig 1). However, this strong effect is biased, as the general public between both countries barely differ, d = 0.15 (because of the large sample size the difference is still significant: *t*[2826] = 3.81, *p* = .0001, see Fig 1).

Overall, this indicates that we cannot generalize from student samples to the general public, because they differ randomly across countries and variables, based on our current knowledge. These findings are also relevant, as they provide limitations with regard to the replicability of psychological research and may also explain failed replication. Take the variable ‘perceived respect towards elderly’ as an example. The difference between the student samples and general public was, even in industrialized countries such as Australia, Germany, New Zealand, and the USA, highly inconsistent (Fig 3). The results from New Zealand, for example, failed to replicate in any of the three other countries.

In sum, the failure to replicate psychological findings may not only be due to low power, questionable research practices such as p-hacking, HARKing, or publication bias [12,30–32], but also because effects of context were not considered in the replication.

## Supporting Information

S1 Data Data set. (SAV)

S1 File R code to reconstruct the analyses. (R)
